# Association between circulating levels of ACE2-Ang-(1–7)-MAS axis and ACE2 gene polymorphisms in hypertensive patients

**DOI:** 10.1097/MD.0000000000003876

**Published:** 2016-06-17

**Authors:** Dan Liu, Yongyue Chen, Ping Zhang, Jiuchang Zhong, Lijun Jin, Caojin Zhang, Shuguang Lin, Shulin Wu, Huimin Yu

**Affiliations:** a Department of Cardiology, Guangdong Cardiovascular Institute, Guangdong General Hospital, Guangdong Academy of Medical Sciences, Zhongshan Road No. 2, Yuexiu District, Guangzhou, Guangdong, P.R. China; b State Key Laboratory of Medical Genomics & Shanghai Institute of Hypertension, Ruijin Hospital Affiliated to Shanghai Jiao Tong University School of Medicine, Shanghai, P.R. China.

**Keywords:** angiotensin-converting enzyme 2, angiotensin-(1–7), enzyme-linked immunosorbent assay, essential hypertension, polymerase chain reaction, polymorphisms, renin–angiotensin–aldosterone system, single nucleotide

## Abstract

The angiotensin-converting enzyme 2-angiotensin-(1–7)-MAS axis (ACE2-Ang-[1–7]-MAS axis) plays an important role in the control of blood pressure. Some previous studies indicated that the genetic variants of ACE2 may have a potential to influence this axis. Therefore, the present study aimed at examining the association of ACE2 polymorphisms with circulating ACE2 and Ang-(1–7) levels in patients with essential hypertension.

Hypertensive patients who met the inclusion criteria were enrolled in the present study. Three Tag single-nucleotide polymorphisms (rs2106809, rs4646155, and rs879922) in ACE2 gene were genotyped for all participants. Circulating ACE2 and Ang-(1–7) levels were detected by enzyme-linked immunosorbent assay.

There were 96 (53.0%) females and 85 (47.0%) males participating in the present study. The circulating Ang-(1–7) levels were significantly greater in female patients carrying the rs2106809 CC or CT genotype compared with those carrying the TT genotype (1321.9 ± 837.4 or 1077.5 ± 804.4 pg/mL vs 751.9 ± 612.4 pg/mL, respectively; *P* = 0.029, analysis of variance), whereas the circulating Ang-(1–7) levels were comparable among genotypes in male patients. In addition, there was no significant difference in the circulating ACE2 levels among rs2106809 CC, CT, and TT genotype groups in both female and male patients. The circulating ACE2 and Ang-(1–7) levels were related to neither rs4646155 nor rs879922 in female or male patients.

In conclusion, the rs2106809 polymorphism of the ACE2 gene may be a determinant of the circulating Ang-(1–7) level in female patients with hypertension, suggesting a genetic association between circulating Ang-(1–7) levels and ACE2 gene polymorphisms in patients with hypertension.

## 1 Introduction

Essential hypertension (EH) is one of the major risk factors for cardiovascular disease. The pathophysiology of EH is very complex and involves many factors. The impaired capacity of the kidney to excrete sodium in response to elevated blood pressure increases the vulnerability to hypertension, irrespective of the initiating cause.^[1]^ Endothelial dysfunction is involved in the development and progression of EH through increasing the vascular tone.^[2]^ The renin–angiotensin–aldosterone system (RAAS) plays a crucial role in the pathogenesis and progression of EH. In the RAAS, angiotensin-converting enzyme (ACE) converts angiotensin I (Ang I) into angiotensin II (Ang II), which binds to Ang II type 1 receptor (AT1R) to constrict blood vessels and elevate blood pressure.^[3,4]^ The classical RAAS axis, called ACE–Ang II–AT1R axis, is composed by ACE, Ang II, and AT1R.

In recent years, a counter-regulatory axis of the RAAS, angiotensin-converting enzyme 2-angiotensin-(1–7)-MAS axis (ACE2-Ang-[1–7]-MAS axis) has been discovered and has been proven to counterbalance the adverse actions of the classical RAAS axis. ACE2 cleaves Ang II to generate Ang-(1–7), which is one of the main effector peptides in ACE2-Ang-(1–7)-MAS axis. Ang-(1–7) counteracts the effects of Ang II and dilates blood vessels to lower blood pressure.^[5–10]^ ACE2 regulates the RAAS and blood pressure through the degradation of Ang II and the formation of Ang-(1–7).^[11]^

Some previous studies suggested that genetic variants in the ACE2 gene might have a potential to affect ACE2 or Ang-(1–7) level in the human body. In the Leeds Family Study, ACE, ACE2, and neutral endopeptidase (NEP) activities were measured in plasma from 534 subjects and it was indicated that up to 67% of the phenotypic variation in circulating ACE2 could be accounted for by genetic factors.^[12]^ Some genotype association studies indicated that single-nucleotide polymorphisms (SNPs) in the ACE2 gene were related with cardiovascular diseases. A clinical study of 3408 patients found that ACE2 rs2106809 T allele conferred a 1.6-fold risk for hypertension in Chinese women.^[13]^ An Indian study confirmed this finding, and it found that ACE2 rs2106809 polymorphism was associated with EH in both females and males.^[14]^ Lieb et al reported that ACE2 gene polymorphisms rs4646156, rs879922, rs4240157, and rs233575 might be associated with left ventricular mass, septal wall thickness, and left ventricular hypertrophy in hemizygous men.^[15]^ All the studies above provided strong evidence that ACE2 gene polymorphisms might have effects on cardiovascular system. We hypothesized that the effects of ACE2 gene polymorphisms on EH were mediated by regulating ACE2 and Ang-(1–7) levels. Therefore, the present study was carried out to investigate whether ACE2 gene polymorphisms could influence the circulating levels of ACE2-Ang-(1–7)-MAS axis in Chinese Han patients with EH.

## 2 Methods

### 2.1 Study population

In this prospective study, Chinese Han patients who met the following criteria were included: age 18 to 79 years; a history of essential hypertension; and diastolic blood pressure (DBP) 90 to 109 mm Hg or systolic blood pressure (SBP) 140 to 179 mm Hg. The exclusion criteria were shown as follows: secondary hypertension, including idiopathic hyperaldosteronism, renal artery stenosis, and so on; the use of drugs that may influence RAAS hormone levels, such as direct renin inhibitors (DRIs), ACE inhibitors (ACEIs), Ang-II receptor blockers (ARBs), or aldosterone antagonists (AAs); any clinically important abnormal laboratory finding, such as alanine aminotransferase (ALT) or creatinine that was more than twice the upper limit of normal; pregnant or lactating females; a history or suspicion of alcohol or drug abuse; and mental illness. The study complies with the Declaration of Helsinki. All procedures were reviewed and approved by the local Institutional Review Board, and written informed consents were obtained from all the participants.

### 2.2 Evaluation of clinical and biochemical parameters

Blood pressure and heart rate were measured by trained doctors or nurses using an electronic sphygmomanometer after the patient had rested for at least 10 minutes in a seated position and were determined as the mean of 3 measurements taken 1 minute apart. Biochemical parameters were measured using standard procedures in the hospital clinical laboratory.

### 2.3 Collection and preservation of blood samples

Blood samples were collected from participants by trained nurses at 8:00 a.m. Then the samples were sent to laboratory for centrifugation (1500*g*, 20 minutes). Serum, plasma, and blood cells were separated and stored in a deep freezer (−80°C) for later use. Avoid freeze/thaw cycles to keep them stable.

### 2.4 Measurement of circulating ACE2 and Ang-(1–7) level

Circulating ACE2 and Ang-(1–7) level were measured using commercial enzyme-linked immunosorbent assay (ELISA) kits (Cloud-Clone Corp, Houston). The ELISA for measurement of ACE2 employed the sandwich enzyme immunoassay technique and the ELISA for measurement of Ang-(1–7) employed the competitive inhibition enzyme immunoassay. All procedures were conducted rigorously in accordance with the instructions in the kits. The optical density values were obtained by a microplate reader at a wavelength of 450 ± 10 nm. The sample concentrations were calculated from standard curve created by plotting the mean absorbance obtained for each reference standard against its concentration.

### 2.5 SNP selection and genotyping

Three Tag SNPs (rs2106809, rs4646155, and rs879922) at various allele frequencies were selected from a subset of SNPs of ACE2 with a minor allele frequency equal to or greater than 3% in the HapMap CHB (Han Chinese in Beijing) database by using the pair-wise option of the Haploview version of the Tagger program (http://www.broad.mit.edu/mpg/haploview). An *r*^2^ value of 0.8 was selected as a threshold for all analyses.^[13]^ Genomic DNA was extracted from peripheral venous blood leukocytes using standard procedures. The polymerase chain reaction (PCR) was used to amplify the gene regions. PCR primers and single base extension were designed by SEQUENOM MassARRAY Assay Design v4.0 software. The primer pairs used were as follows: rs2106809; forward primer: 5′-ACGTTGGATGGAGAGAACTTTGGAAACCTG-3′; reverse primer: 5′- ACGTTGGATGGCTGCTGATGTAGAAGTGTG-3′; rs4646155; forward primer: 5′-ACGTTGGATGGGCATGTTCTTAACCTTGGC-3′; reverse primer: 5′-ACGTTGGATGCCAATATGACCCTGTAAACC-3′; rs879922; forward primer: 5′-ACGTTGGATGGGCAGTTTATTGTACATTGTG-3′; reverse primer: 5′-ACGTTGGATGGCTCCAGCAAATTCAAGGAC-3′.

The amplified PCR product was purified by Wizard PCR Preps DNA Purification Resin (Promega) and then sequenced using the BIG DYE dideoxy-terminator chemistry (Perkin Elmer) on an ABI 3100 DNA sequencer.

### 2.6 Statistical analysis

Males and females were analyzed separately because the ACE2 gene is located on the X chromosome. Continuous data are presented as mean ± standard deviation (SD). Differences between groups were tested by chi-square test for qualitative parameters and by 1-way analysis of variance (ANOVA) for quantitative parameters. Allele frequencies were calculated from the genotypes of all subjects. The Hardy–Weinberg equilibrium (HWE) was assessed by chi-square analysis. The association of circulating ACE2 and Ang-(1–7) levels with SBP and DBP was analyzed using univariate linear regression model. A 2-tailed *P* value of <0.05 was considered statistically significant. All analyses were performed using SPSS statistical software (Version 13.0; SPSS, Chicago, IL).

## 3 Results

A total of 181 patients were enrolled in the present study, including 96 (53.0%) female patients and 85 (47.0%) male patients. ACE2 genotypes, circulating ACE2, and Ang-(1–7) levels were determined for all patients. The rs4646155 and rs879922 were found to be in complete linkage disequilibrium in both female and male patients. Table 1 displays the baseline characteristics of females and males. Tables 2–5 show the ACE2 genotype distribution, and clinical and biochemical parameters of the patients. Tables 6 and 7 show the comparison of circulating ACE2 and Ang-(1–7) levels among genotype groups. Table 8 displays the effects of circulating ACE2 and Ang-(1–7) levels on both SBP and DBP.

**Table 1:**
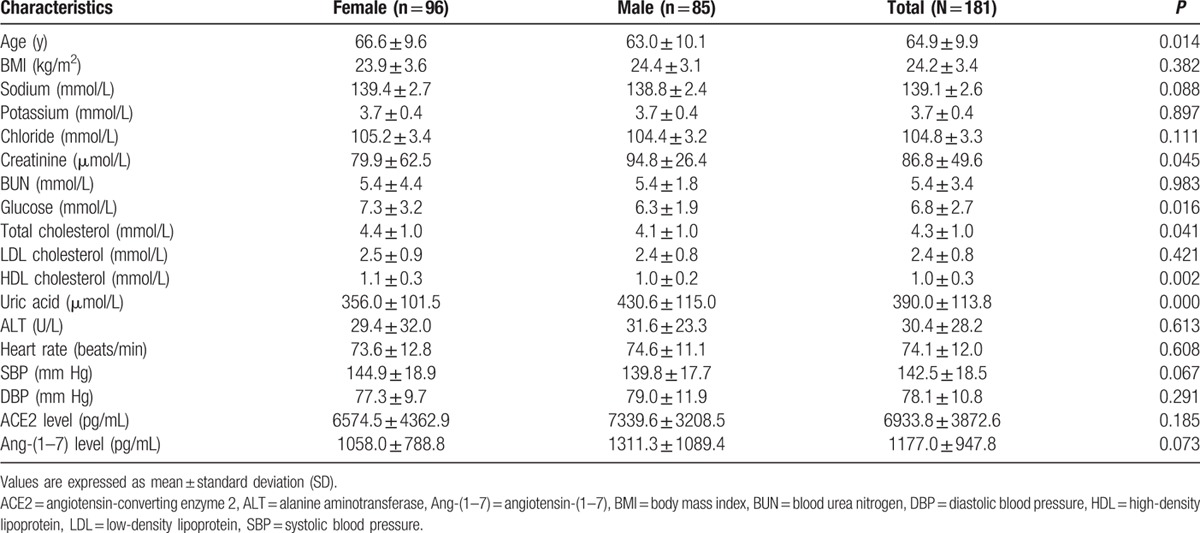
Baseline characteristics of the study population.

**Table 2:**
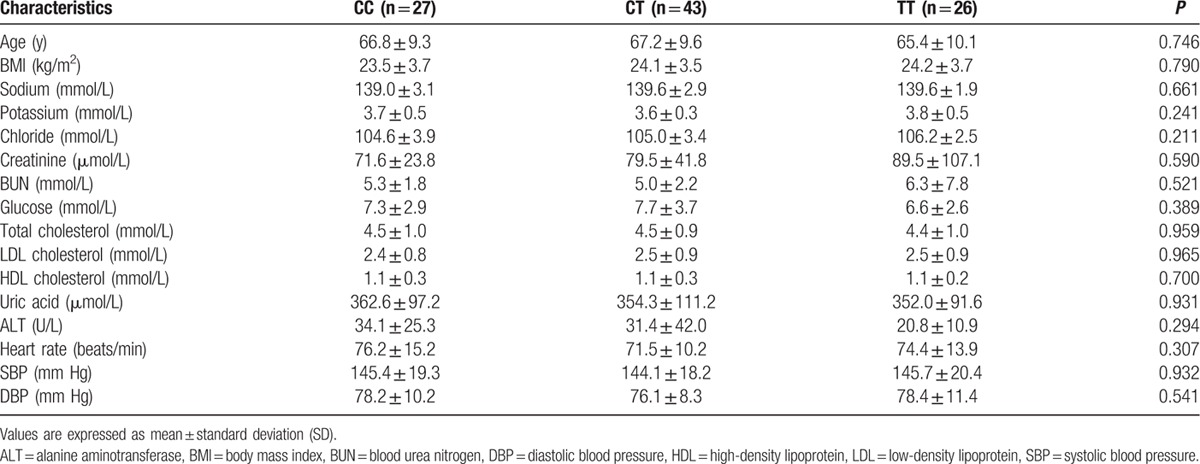
Clinical and biochemical characteristics of female patients by ACE2 rs2106809 genotype.

**Table 3:**
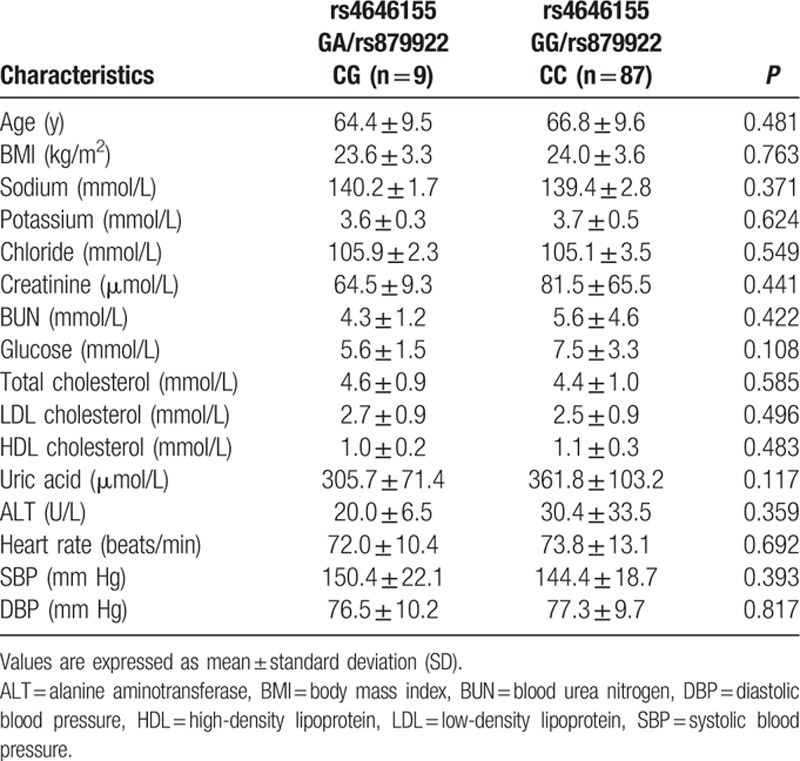
Clinical and biochemical characteristics of female patients by ACE2 rs4646155 or rs879922 genotype.

**Table 4:**
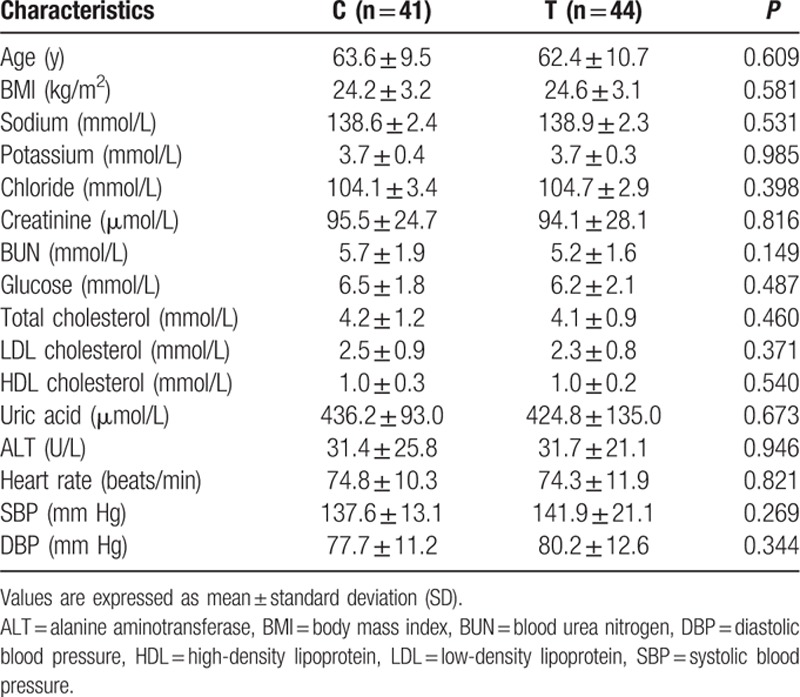
Clinical and biochemical characteristics of male patients by ACE2 rs2106809 genotype.

**Table 5:**
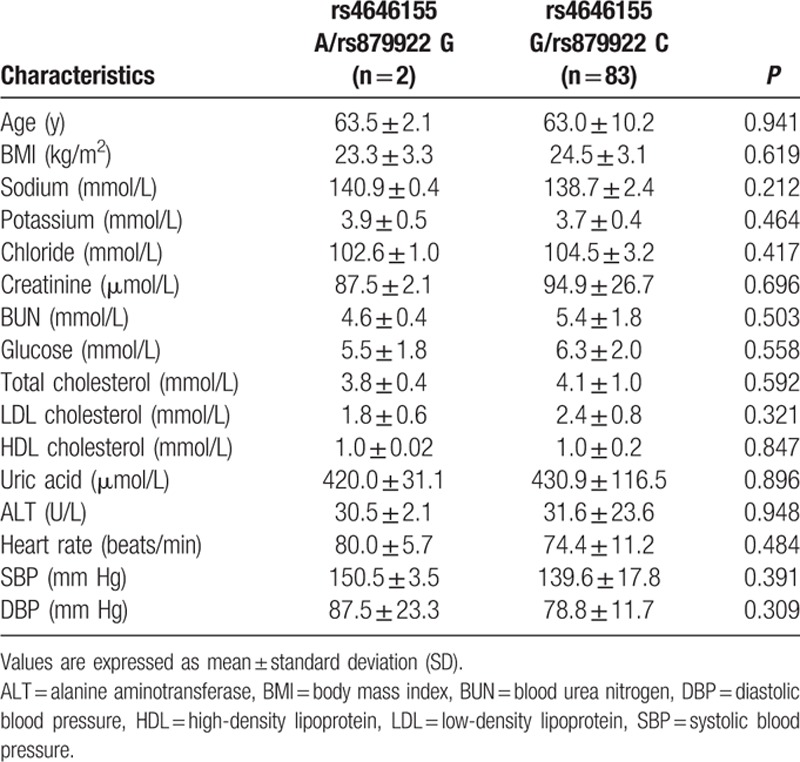
Clinical and biochemical characteristics of male patients by ACE2 rs4646155 or rs879922 genotype.

**Table 6:**
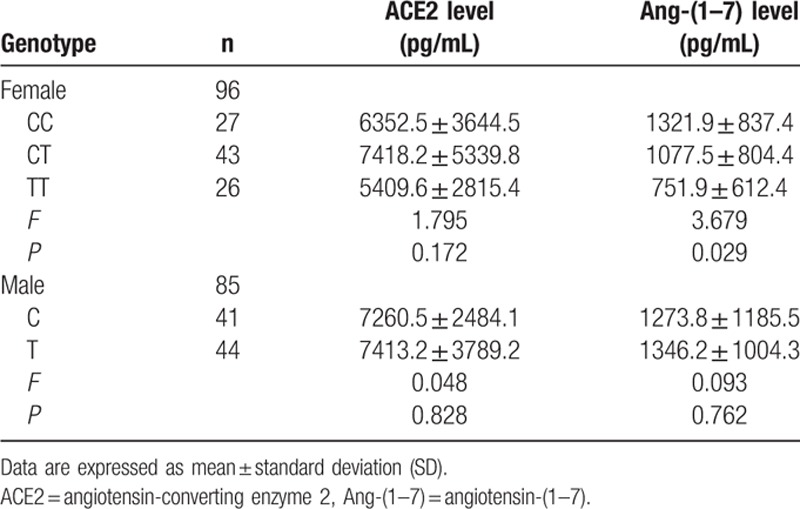
Circulating ACE2 levels and Ang-(1–7) levels in patients according to the ACE2 rs2106809 genotype.

**Table 7:**
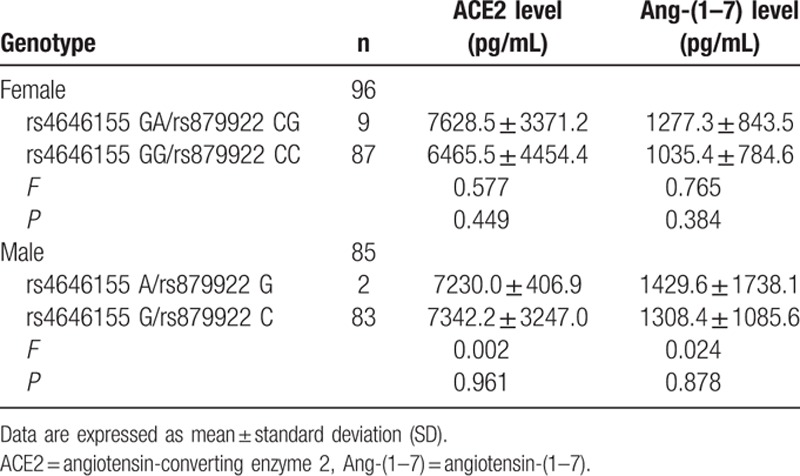
Circulating ACE2 levels and Ang-(1–7) levels in patients according to the ACE2 rs4646155 or rs879922 genotype.

**Table 8:**
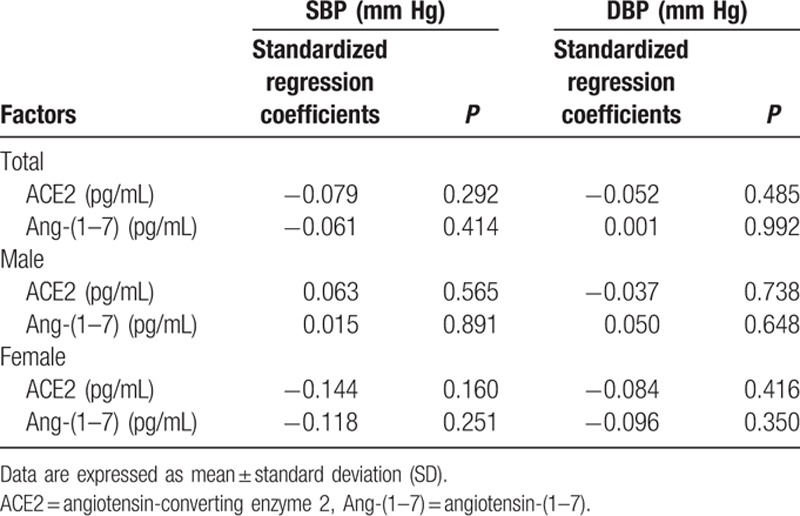
Associations of circulating ACE2 and Ang-(1–7) levels with SBP and DBP according to univariate regression analyses.

### 3.1 Association of circulating ACE2 and Ang-(1–7) levels with ACE2 genotypes in female patients

The CC, CT, and TT genotypes of ACE2 rs2106809 were present in 27 patients (28.1%), 43 patients (44.8%), and 26 patients (27.1%), respectively. Allele frequencies were 50.5% for the C allele and 49.5% for the T allele. The genotype frequencies were in HWE (*χ*^2^ = 1.040, df = 1, *P* = 0.308). Baseline characteristics were comparable among rs2106809 genotypes (Table 2). The circulating Ang-(1–7) levels were significantly greater in patients carrying the rs2106809 CC or CT genotype compared with those carrying the TT genotype (1321.9 ± 837.4 or 1077.5 ± 804.4 pg/mL vs 751.9 ± 612.4 pg/mL, respectively; *P* = 0.029, ANOVA) (Table 6). There was no significant difference in circulating ACE2 levels among rs2106809 genotype groups (Table 6).

The ACE2 rs4646155 GA or rs879922 CG genotype was observed in 9 patients (9.4%), and the rs4646155 GG or rs879922 CC genotype was observed in 87 patients (90.6%). Allele frequencies were 4.7% for the rs4646155 A or rs879922 G allele and 95.3% for the rs4646155 G or rs879922 C allele. The genotype frequencies were in HWE (*χ*^2^ = 0.232, df = 1, *P* = 0.630). Baseline characteristics were comparable among genotype groups (Table 3). There was no significant difference in the circulating ACE2 and Ang-(1–7) levels among rs4646155 or rs879922 genotype groups (Table 7).

### 3.2 Association of circulating ACE2 and Ang-(1–7) levels with ACE2 genotypes in male patients

The C and T genotype of ACE2 rs2106809 were present in 41 patients (48.2%) and 44 patients (51.8%), respectively. Baseline characteristics were comparable among genotype groups (Table 4). There was no significant difference in the circulating ACE2 and Ang-(1–7) level between rs2106809 C genotype and T genotype group (Table 6).

The ACE2 rs4646155 A or rs879922 G genotype was observed in 2 patients (2.4%) and the rs4646155 G or rs879922 C genotype was observed in 83 patients (97.6%). Baseline characteristics were comparable among genotype groups (Table 5). There was no significant difference in the circulating ACE2 and Ang-(1–7) levels among rs4646155 or rs879922 genotype groups (Table 7).

### 3.3 Association of circulating ACE2 and Ang-(1–7) levels with SBP and DBP

To investigate the impacts of ACE2 and Ang-(1–7) levels on SBP and DBP, we performed an analysis using univariate linear regression model. Nevertheless, we could not find any significant relationship of circulating ACE2 and Ang-(1–7) levels with SBP and DBP in both female and male patients (Table 8).

## 4 Discussion

Several previous studies suggested that genetic variants in the ACE2 gene might have effects on EH and ACE2 or Ang-(1–7) levels.^[13,14]^ Therefore, the present study was carried out to address this issue. As far as we know, it is the first clinical study to investigate the impacts of ACE2 polymorphisms on circulating ACE2 and Ang-(1–7) levels. Whats more, the present study found a significant relationship between ACE2 rs2106809 and circulating Ang-(1–7) levels, and confirmed our previous speculation.

We found that in female patients, circulating Ang-(1–7) levels was significantly higher in ACE2 rs2106809 CC or CT genotype than TT genotype. In the previous studies, Fan et al^[13]^ found that ACE2 rs2106809 T allele (TT + CT genotype) was a contributor to hypertension in women, and Patnaik et al^[14]^ found that ACE2 rs2106809 TT genotype is an independent risk factor for hypertension in Indian women. It is apparent that our finding can account for the previous studies perfectly. ACE2 rs2106809 TT genotype might be able to lower the circulating Ang-(1–7) levels through down-regulating ACE2 gene expression or decreasing ACE2 activity or other pathways. Besides, Ang-(1–7) exerts a variety of beneficial effects on cardiovascular system and plays a protective role in EH.^[5–10]^ Therefore, ACE2 rs2106809 TT genotype might increase the susceptibility to EH by lowering Ang-(1–7) level.

In female patients, ACE2 rs2106809 CC or CT genotype carriers had higher circulating ACE2 levels than TT genotype carriers, but the difference failed to reach a significant level. This phenomenon is intriguing. ACE2 converts Ang II into Ang-(1–7), so it is at least one of the determinants of Ang-(1–7) level.^[5–10]^ Moreover, rs2106809 is located at ACE2 gene and it might influence Ang-(1–7) level though regulating ACE2 level. It would be more reasonable that there was a significant difference in circulating ACE2 levels among genotypes. Although the finding seems unreasonable, some possible reasons could still account for it. First of all, the relatively small sample size would result in the failure to reach statistical significance. If the sample size was enlarged, circulating ACE2 level might have a chance to be significantly different among genotypes. Secondly, ACE2 is found not only in human serum, but also in a variety of tissues, including vascular endothelium, intrarenal vessels, renal tubular epithelium, and so on.^[16]^ Whats more, the tissue distribution of ACE2 varies considerably.^[16]^ Thus, we could speculate that ACE2 levels might differ significantly among genotypes in tissues like vascular endothelium or renal tubular epithelium, and then induce the difference in Ang-(1–7) levels among genotypes. Last but not the least, there is likely to be linkage disequilibrium between ACE2 rs2106809 and the functional SNPs in the genes for other enzymes that can alter Ang-(1–7) level. Apart from ACE2, Ang-(1–7) level can also be affected by many other enzymes. It can be elevated through its formation by prolylcarboxypeptidase (PRCP),^[17]^ NEP, prolyl endopeptidase (POP), thimet oligopeptidase (TOP),^[18]^ and be reduced through its degeneration by aminopeptidase A.^[19]^ In addition, there might be some unknown enzymes that could affect Ang-(1–7) levels. Therefore, it is reasonable to speculate that some functional SNPs in the genes for these enzymes might be in linkage disequilibrium with ACE2 rs2106809 and be responsible for the difference in Ang-(1–7) level among genotypes.

We speculated that ACE2 rs2106809 might have effects on the ACE2 levels. Although the difference in ACE2 levels among genotypes did not reach a significant level, the circulating ACE2 levels tend to be greater in CC or CT genotype compared with that in TT genotype. If the effects did exist, the mechanism should be speculated. One of the possible mechanisms can be the microRNA, which could modulate endothelial function via translational repression and/or posttranscriptional degradation.^[20]^ As we stated above, endothelial dysfunction is one of the main contributors to hypertension. In addition, it has been reported that microRNA might regulate RAAS expression via binding to the targeted sites of genes in preeclampsia.^[21]^ Thus, although ACE2 polymorphism rs2106809 is located in intron 1, a noncoding region of ACE2 gene, it is still possible to be in a microRNA-binding site or microRNA gene, and thereby has a potential to regulate gene expression by altering the miRNA–mRNA interaction.^[22]^The second plausible mechanism can be the creation or disruption of a splicing motif, such as enhancer or silencer, and this may alter the splicing efficiency of ACE2.^[23]^ All these possible mechanisms should be explored in the future.

In the present study, there was no significant relationship between circulating ACE2 or Ang-(1–7) levels and blood pressure, though it was previously reported that Ang-(1–7) and ACE2 counteracted the actions of Ang II. Nevertheless, our finding could be reasonable. The RAAS is a complex system and has at least 2 axes, as we mentioned above, to balance each other. The alteration of ACE2-Ang-(1–7)-MAS axis may induce the corresponding change of its counter-regulatory axis to balance the whole system. The effects of ACE2-Ang-(1–7)-MAS axis on blood pressure might be nullified by ACE–Ang II–AT1R axis.

There are several limitations that should be mentioned. Firstly, ACE2 and Ang-(1–7) levels should be assessed not just in human blood, but also in other tissues, especially in vascular endothelium. Vascular endothelial dysfunction is proven to be involved in the pathophysiology of EH and regulated by genetic factor.^[2]^ Thus, it will be better to assess ACE2 and Ang-(1–7) levels in tissues like vascular endothelium. Secondly, the possible mechanisms of actions, such as microRNA and creation or disruption of a splicing motif, should be explored further. Thirdly, the present study did not assess the circulating ACE2 activity while it might differ among ACE2 genotypes. Fourthly, more SNPs should be selected and genotyped to search for some potential SNPs in the genes for other enzymes that can alter Ang-(1–7) level and find out whether these SNPs are in linkage disequilibrium with ACE2 rs2106809. Finally, we did not evaluate the level of ACE–Ang II–AT1R axis simultaneously and could not know whether it was the ACE–Ang II–AT1R axis that nullified the effects of ACE2-Ang-(1–7)-MAS axis on blood pressure.

In conclusion, ACE2 polymorphism rs2106809 may be a determinant of the circulating Ang-(1–7) level in female patients with hypertension, suggesting a genetic association between circulating Ang-(1–7) levels and ACE2 gene polymorphisms in patients with hypertension. It will help us gain a better understanding of the relationship between hereditary factors and EH.

## Acknowledgments

The authors thank Mr Xiangfeng Dou (Department of Epidemiology, Beijing Center for Disease Prevention and Control, Beijing 100013, PR China) for his help in statistical analysis.
